# Wearable Pressure Sensor Using Porous Natural Polymer Hydrogel Elastomers with High Sensitivity over a Wide Sensing Range

**DOI:** 10.3390/polym15122736

**Published:** 2023-06-19

**Authors:** Fan Xiao, Shunyu Jin, Wan Zhang, Yingxin Zhang, Hang Zhou, Yuan Huang

**Affiliations:** 1 School of Microelectronics Science and Technology, Sun Yat-Sen University, Guangzhou 510275, China; 2 Hefei National Research Center for Physical Sciences at the Microscale, University of Science and Technology of China, Hefei 230026, China; 3 School of Electronic and Computer Engineering, Peking University Shenzhen Graduate School, Shenzhen 518055, China

**Keywords:** pressure sensor, porous hydrogels, natural polymer

## Abstract

Wearable pressure sensors capable of quantifying full-range human dynamic motionare are pivotal in wearable electronics and human activity monitoring. Since wearable pressure sensors directly or indirectly contact skin, selecting flexible soft and skin-friendly materials is important. Wearable pressure sensors with natural polymer-based hydrogels are extensively explored to enable safe contact with skin. Despite recent advances, most natural polymer-based hydrogel sensors suffer from low sensitivity at high-pressure ranges. Here, by using commercially available rosin particles as sacrificial templates, a cost-effective wide-range porous locust bean gum-based hydrogel pressure sensor is constructed. Due to the three-dimensional macroporous structure of the hydrogel, the constructed sensor exhibits high sensitivities (12.7, 5.0, and 3.2 kPa^−1^ under 0.1–20, 20–50, and 50–100 kPa) under a wide range of pressure. The sensor also offers a fast response time (263 ms) and good durability over 500 loading/unloading cycles. In addition, the sensor is successfully applied for monitoring human dynamic motion. This work provides a low-cost and easy fabrication strategy for fabricating high-performance natural polymer-based hydrogel piezoresistive sensors with a wide response range and high sensitivity.

## 1. Introduction

Wearable pressure sensors capable of detecting human body movements are crucial in muscle motion analysis, speech recognition, disease diagnosis, and health monitoring [1,2,3,4,5,6]. To meet diverse demands in different human body movements monitoring applications, such as subtle joint-bending detection and heavy foot tapping monitoring, pressure sensors are desired to possess high sensitivity over a wide sensing range. Wearable pressure sensors have been developed based on several major sensing mechanisms, including piezoresistive [7,8], capacitive [9], and piezoelectric [10] mechanisms. Among these, piezoresistive pressure sensors based on applied pressure transduction in an electrical conductance of sensing materials have been widely studied due to their easy signal acquisition, simple process, and simple circuit integration [11].

Since wearable pressure sensors directly or indirectly contact skin, selecting flexible soft and skin-friendly materials is important. Hydrogels, a kind of soft material consisting of cross-linked networks of hydrophilic polymers in water, have drawn particular interest for their liquid-like transport and solid-like mechanical properties [12]. There are two categories of hydrogels: natural polymer-based hydrogels and synthetic polymer-hydrogels [12]. Natural polymers were widely used for biomedicine and wearable electronics due to their appealing merits of multi-functionality, ease of accessibility, and biocompatibility. Natural polymer-based hydrogels have high biodegradability and biocompatibility, offering a breakthrough in wearable electronics [12,13,14,15]. Several studies reported wearable pressure sensors with natural polymer-based hydrogels, such as the locust bean gum (LBG)-based [16], cellulosic [17], and chitosan-based [18] hydrogels. For example, Shen et al. used a unique kind of cellulose-rich material with high molecular weight which was isolated directly and simply from wood through rapid dissolution in ionic liquid at temperatures above the glass transition of lignin to prepare a macroporous, compressible lignocellulosic hydrogel [17]. The assembled hydrogel sensors possess a wide responsive stress range (above 0.35 MPa) [17]. Yang et al. reported a wearable pressure sensor based on a resilient, anti-fatigue and freezing-tolerant chitosan-poly (hydroxyethyl acrylamide) double-network hydrogel [18]. The wearable sensor exhibited a sensitive and large-range detection capacity, together with long-term stability and wide operating temperature range [18]. We also reported an elastic and biocompatible hybrid network hydrogel by cross-linking LBG, polyvinyl alcohol (PVA), and carbon nanotubes (CNTs) [16]. A pressure sensor with a double-rough surface of LBG-based hydrogel exhibits a high sensitivity (20.5 kPa^−1^) at the low-pressure range (0.1–1 kPa). However, as the cross-linked network structure inside the hydrogels improves their robustness, the pressure sensitivity significantly decreases in a higher-pressure range (2.28 kPa^−1^, 1–10 kPa; 0.24 kPa^−1^, 10–100 kPa). Thus, developing wearable pressure sensors based on elastic natural polymer-based hydrogels capable of detecting a wide pressure range while maintaining high sensitivity remains challenging.

Many reports have shown that porous structure inside elastomers can effectively distribute the applied pressure, thus increasing the sensing range of wearable pressure sensors and improving their sensitivity at high-pressure ranges [6,19,20,21,22]. Porous elastomers are prepared by the sacrificial template method by mixing elastomer solution with templates, such as salt, sugar, and citric acid monohydrate, and dissolving the mixture in water after solidifying [6,19,22,23,24,25]. For example, Sang et al. reported a porous composite foam via a simple heat molding of conductive fillers and elastomer together with commercially available popcorn salts followed by water-assisted salt removal [6]. Zhao et al. reported a multilayered and graded-porosity polydimethylsiloxane/silver nanoparticle sponge, which can be fabricated using the sacrificial template method of mixing polydimethylsiloxane solution with citric acid monohydrate templates [19]. Lo et al. reported an elastomeric sponge-based sensor based on a porous polydimethylsiloxane sponge, which was fabricated from a sugar cube sacrificial template [24]. However, salt, sugar, and citric acid monohydrate are soluble in hydrogels; thus, they are unsuitable for porous natural polymer-based hydrogels. Consequently, developing porous natural polymer-based hydrogels using a suitable and economic sacrificial template is highly desired for wearable pressure sensors.

In this study, a highly porous LBG-based hydrogel is synthesized from commercially available rosin particles as the sacrificial template. These particles are insoluble in water but can be leached in ethanol. For an optimized porosity of the LBG-based hydrogel, the wearable pressure sensor based on porous LBG-based hydrogels sandwiched between two carbon cloth electrodes can monitor a wide range of pressure with high sensitivities (12.7, 5.0, and 3.2 kPa^−1^ under 0.1–20, 20–50, and 50–100 kPa). The proposed sensor’s sensitivities at the high-pressure range are superior to those of the previous hydrogel sensors. The sensor’s wide pressure range with high sensitivities is ascribed to a three-dimensional macroporous structure with dense large-sized pores (~100–150 μm). The sensor also offers a fast response time (263 ms) and good durability over 500 loading/unloading cycles. Using the sensor for various human movement applications is demonstrated as a proof-of-concept. A low-cost and facile craft route to fabricate highly porous natural polymer-based hydrogels is provided, which can be useful for high-performance wearable pressure sensor applications.

## 2. Materials and Methods

### 2.1. Preparation of Porous LBG-Based Hydrogels and Original LBG-Based Hydrogels

Porous LBG-based hydrogels were prepared by mixing 4 g PVA (1750 ± 50, Shanghai Yuanye Bio-Technology Co., Ltd., Shanghai, China), 300 mg CNTs (XFNANO Materials Tech Co., Ltd., Nanjing, China), and 40 mL deionized water. The mixture was heated to 100 °C under vigorous stirring until fully dissolved PVA. Then 1 g LBG (Aladdin, Shanghai, China) was added to the mixture. The rosin particles and the mixture solution were stirred at specific ratios until reaching homogeneous dissolution. The mixture was transferred to an environmental cabinet at −20 °C for 12 h to form LBG/PVA/CNTs/rosin hydrogels. The hydrogels were immersed in ethanol for 24 h to dissolve rosin particles.

Original LBG-based hydrogels were prepared by mixing 4 g PVA (1750 ± 50, Shanghai Yuanye Bio-Technology Co., Ltd.), 300 mg CNTs (XFNANO Materials Tech Co., Ltd.), and 40 mL deionized water. The mixture was heated to 100 °C under vigorous stirring until fully dissolved PVA. Then 1 g LBG (Aladdin) was added to the mixture. The mixture was transferred to an environmental cabinet at −20 °C for 12 h to form LBG/PVA/CNTs hydrogels.

### 2.2. Materials Characterization

The hydrogels’ microstructure was examined by a scanning electron microscope (SEM, Zeiss Crossbeam 350 Germany). The porosities of the porous hydrogels was examined by an X-ray microscope (XRM, Zeiss, Xradia 520 Versa, Germany). In brief, 3D CT images of the hydrogels were obtained by XRM and the porosities of the porous hydrogels can be identified in the 3D CT images by different contrast automatically by Dragonfly Pro software. The compressive mechanical properties of porous hydrogels were tested by ZQ-990LB (China) equipped with a force gauge (maximum force: 100 N). The compressive tests were performed on a cylindrical sample (diameter: 1 cm, height: 4.13 mm) at 10 mm min^−1^. The weight retention test was conducted at 25 °C and 15% humidity.

### 2.3. Electrical Characterization

Hydrogels were tailored into 1 cm × 1 cm square pieces. The sensors with hydrogels sandwiched between two carbon cloth electrodes are fabricated. To evaluate the pressure sensing performance, a testing machine ZQ-990LB equipped with a force gauge (maximum force: 100 N) was used to apply pressure to the device, and the corresponding current signals were collected by a digital source meter (Keithley 2635B, America) at the working voltage of 1.5 V.

## 3. Results and Discussion

### 3.1. Fabrication and Characterization of Porous and Original Hydrogels

**Figure 1:**
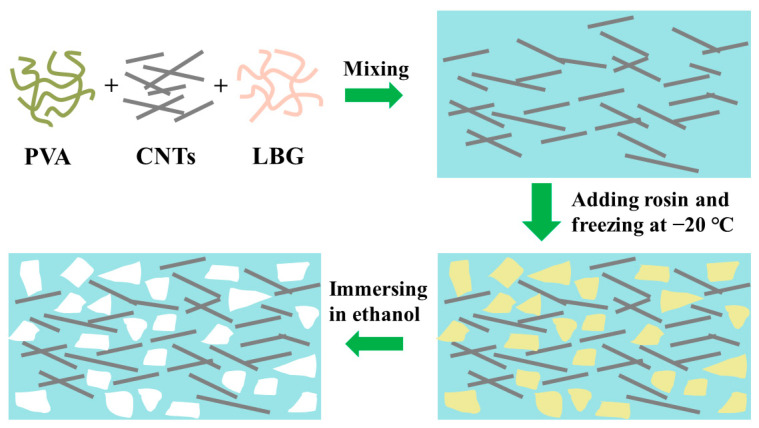
Schematic illustrations of the porous hydrogels’ fabrication process.

The fabrication process of porous hydrogels is schematically illustrated in Figure 1. The processes start with PVA and CNTs dissolved in deionized water, followed by the addition of LBG. This mixture solution and rosin particles were mixed under continuous stirring until reaching homogeneous dispersion. The hydrogels were formed by freezing the mixture. Since the rosin particles were barely soluble in ethanol, their amount in the hydrogels was well preserved. Removing the rosin particles with ethanol resulted in a highly porous structure with pore sizes similar to the grain size of rosin particles. The procedure involved a simple hand mixing of LBG, CNTs, and PVA with low-cost rosin in non-toxic solvents. Based on different grain sizes of rosin particles, porous structures with various geometries can be achieved, which may influence the mechanical and piezoresistive properties of the hydrogels. Here, common commercial rosin particles with sizes of 100–150 μm (Figure S1, Supporting Information) were used as an example to demonstrate the method’s feasibility. For comparison, original hydrogels were also prepared by a similar method without introducing rosin particles. The major interactions in the hydrogels are illustrated in our previous work [16]. The high mechanical strength and elasticity of PVA made it a useful primary polymer network. The LBG and PVA molecules containing large numbers of hydrogen bonds directly formed intra- and inter-hydrogen bonds. CNTs were essential for constructing a conductive 3D network.

**Figure 2:**
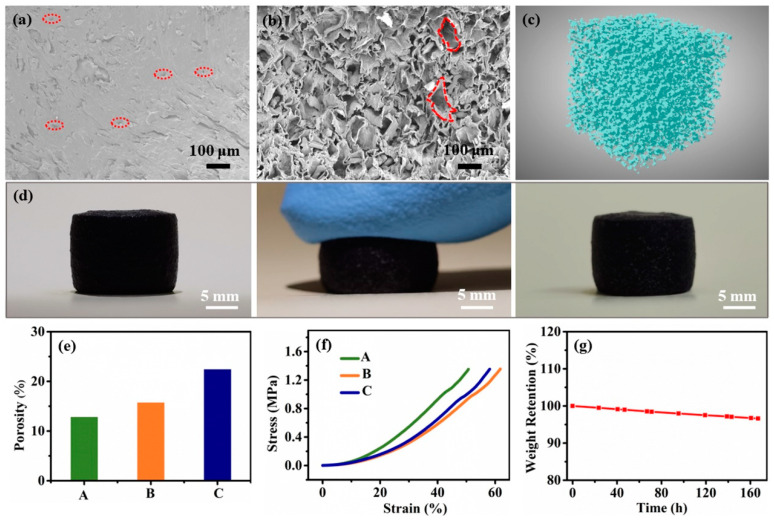
Cross-sectional SEM images of (**a**) original and (**b**) porous hydrogels. Small pores (sub 10 μm level) are marked with red circle in (**a**). Large pore (100–150 μm) are marked with red circle in (**b**). (**c**) X-ray microtomography of porous hydrogels. (**d**) The compressive recovery behavior of porous hydrogels. (**e**) The porosity of hydrogel A, B, and C. (**f**) Compressive stress–strain curves of all three hydrogel samples. (**g**) Weight variation of hydrogel B at 25 °C and 15% humidity.

The microstructure of the hydrogels is observed by SEM (Figure 2a,b). A few cracks and small pores (sub 10 μm level, red circle) are visible in the cross-sectional images of the original hydrogels (Figure 2a). In contrast, the porous hydrogels show a different porous morphology with large pore sizes (100–150 μm) agree with the grain size of rosin particles (Figure 2b). XRM evaluates smaller micropore sizes in porous hydrogels. The tomography of 100 × 100 × 100 μm^3^ highlights three-dimensional morphological features of porous hydrogels and the sub 10 μm level pores (Figure 2c). The SEM and XRM results suggest that both sub 10 μm level pores and 100–150 μm level pores are inside the porous hydrogel. Due to three-dimensional macroporous structures, the porous hydrogels demonstrated promising compressive elasticity that can be compressed and recovered to the original shape after releasing the compressive force (Figure 2d).

The weight fraction of rosin particles in the mixture (LBG, PVA, CNTs, deionized water, and rosin particles) essentially controls the porosities of the porous hydrogel, where rosin particles are dissolved after soaking in ethanol solution. XRM is also employed to evaluate the porous hydrogels’ porosity. Figure 2e shows a concomitant increase in porosity with the weight fraction of rosin particles: 12.8% for the hydrogel with 0.5% weight fraction of rosin particles (denoted as hydrogel A), 15.7% for the hydrogel with 1.5% weight fraction of rosin particles (denoted as hydrogel B), and 22.4% for the hydrogel with 3.0% weight fraction of rosin particles (denoted as hydrogel C).

To investigate the influence of porosity on mechanical softness, typical stress–strain curves of porous hydrogels from compression are depicted in Figure 2f. The higher porosity suggests that more pores are closed under pressure, and stiffness decreases. Thus, when the porosity increases from 12.8% to 15.7%, the compressive strain at a compressive stress of 1.35 MPa increases from 50.7% for hydrogel A to 61.8% for hydrogel B. However, when the porosity increases to 22.4%, the compressive strain of hydrogel C decreases to 58.1%, attributed to the residual rosin particles in hydrogel C (Figure S2, Supporting Information), which are not completely removed by ethanol. 

Moreover, the long-term stability of hydrogels is still a challenge to be addressed. The weight retention of hydrogel B was measured with the weight ratio of W_t_ (weight of hydrogel B at time t) and W_0_ (the initial weight of hydrogel B) at 25 °C. As shown, hydrogel B maintains approximately 96.6% of its initial weight after 7 days of storage, indicating the desirable durability of hydrogel for long-term use (Figure 2g).

### 3.2. Sensing Performance of the Sensors

The three-dimensional macroporous structure of porous hydrogels using rosin particles as the sacrificial template demonstrates exceptional compressive elasticity and a high-compressive strain, enabling their potential application as piezoresistive pressure sensors. The piezoresistive sensors with original or porous hydrogels sandwiched between two carbon cloth electrodes were fabricated and investigated under compressive pressure stimuli. The sensitivity (*S*) is defined as *S* = δ((*I* − *I*_0_)/*I*_0_)/δ*P*, where *I*_0_ is the baseline current under no pressure, *I* is the responsive current under applied pressure, and *P* is the applied pressure [16]. 

**Figure 3:**
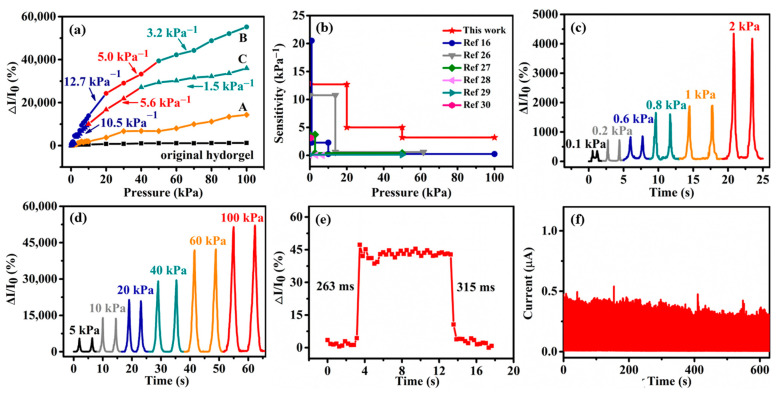
(**a**) The relative current changes for the sensors with original hydrogels and hydrogels A, B, and C; the slope of the curve indicates sensitivity. (**b**) Detection range and sensitivity of our sensor with hydrogel B in comparison with other piezoresistive sensors based on hydrogels as reported in references [16,26,27,28,29,30]. (**c**) The relative current changes for the sensor with hydrogel B under low serial pressures (0.1–2 kPa). (**d**) The relative current changes for the sensor with hydrogel B under high serial pressures (5–100 kPa). (**e**) The sensor’s response time and recovery time with hydrogel B. (**f**) Stability of the sensor with hydrogel B at 3 kPa for 500 cycles.

The response curves of sensors are plotted in Figure 3a. Comparing the sensors with original hydrogels and porous hydrogels reveals that the sensor with original hydrogels exhibits a relatively low current change in response to pressure. The improved sensing performance of porous hydrogel sensors (hydrogels A, B, C) suggests that the macroporous hydrogel structure endows the sensor to respond to pressure more effectively. Moreover, the sensor’s sensitivity and measurement range depend on the hydrogels’ porosity. Compared with the sensor with hydrogel A (12.8% porosity), the sensor with hydrogel B (15.7% porosity) exhibits a greater current change in response to pressure. The higher porosity could further increase the current by forming more current paths with increased pressure. However, the monotonous increase in the porosity of a porous structure cannot automatically improve the sensitivity. The lower sensitivity of the sensor with hydrogel C (22.4% porosity) is observed compared with that of the sensor with hydrogel B (15.7% porosity). This is because residual rosin particles increase gradually with increased rosin content (Figure S2, Supporting Information). Due to the residual rosin particles, the stiffness of hydrogel C increases (Figure 2f), generating fewer current paths under the same pressure as hydrogel B. Thus, the complete dissolution of rosin particles and the formation of an effective porous structure is formed from 1.5% to 3.0% rosin in the mixture (LBG, PVA, CNTs, deionized water, and rosin particles); however, the further addition of rosin particles lowers the sensors’ performance. 

Based on the lowest stiffness of hydrogel B, the sensor monitors pressure more sensitively over the wide pressure range. In the pressure range below 20 kPa, the sensor shows a high sensitivity of 12.7 kPa^−1^. It shows a slightly lower sensitivity of 5.0 kPa^−1^ in the 20–50 kPa pressure range and maintains a 3.2 kPa^−1^ in the 50–100 kPa. Compared to our previously reported sensors (double-rough surface LBG/PVA/CNTs hydrogel) [16], the hydrogel B-based sensor exhibits better sensitivity over a wide linear detection range (Figure 3b). For example, the sensitivity of this sensor at low pressure (12.7 kPa^−1^, 1–20 kPa) is higher than that of a double-rough surface LBG/PVA/CNTs hydrogel-based sensor (2.28 kPa^−1^, 1–10 kPa). The sensitivity detection range of the sensor based on hydrogel B (1–20 kPa) is much wider than that of the double-rough surface hydrogel sensor (1–10 kPa). Furthermore, the sensitivities of this sensor at a high-pressure range (50–100 kPa) are 13.3-fold higher than that of the sensor with double-rough surface LBG/PVA/CNTs hydrogel. These results indicate that porous structure can improve sensitivity and the detection range simultaneously, and it is crucial in the sensor’s response to high pressure. Compared to other hydrogel-based sensors [16,26,27,28,29,30], our sensor’s sensitivities at the high-pressure range are superior to those of the previous hydrogel sensors (Figure 3b). 

Due to its high sensitivity and wide sensing range, the hydrogel B-based sensor was selected for subsequent experiments. To verify the reliability of the sensor, different dynamic pressures were applied. Figure 3c,d shows that the increased applied pressure simultaneously raises the current response. Hence, the sensor can accurately distinguish between different levels of force in the pressure range of 0.1–2 kPa (Figure 3c) and 5–100 kPa (Figure 3d). Additionally, it shows a detection limit of 100 Pa. The response/relaxation time of the device was also analyzed. The proposed sensor immediately responds when a key is placed on it. The response and recovery time is 263 and 315 ms, respectively (Figure 3e). Durability is a critical aspect for wearable piezoresistive sensors. In order to test the durability of hydrogel B-based sensor, 500 loading/unloading cycles at 3 kPa and 20 kPa were performed. The results are shown Figure 3f and Figure S3 (Supporting Information), which indicates the long-term durability and stability of hydrogel B-based sensor. We also compared the response time, detection limit, and durability of our sensor and those of the previous hydrogel sensors [16,26,27,28,29,30] (Table S1, Supporting Information). Further studies are needed to improve the response time, detection limit, and durability of our sensor.

### 3.3. The Sensing Mechanism of the Sensor

**Figure 4:**
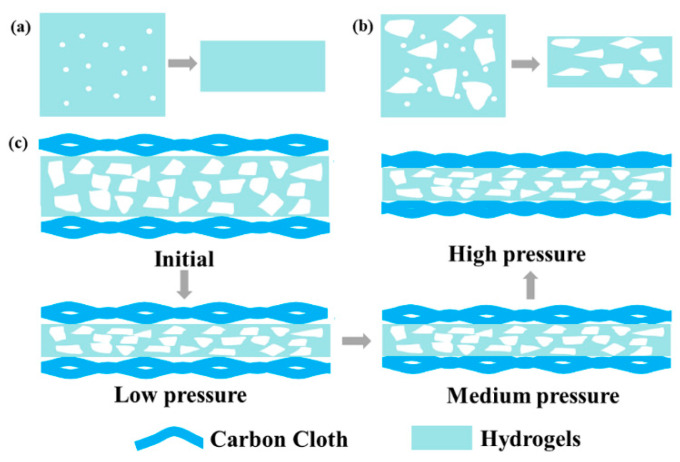
Schematic illustration of the piezoresistive mechanism of (**a**) original and (**b**) porous hydrogels, and (**c**) the sensor structure with porous hydrogels and its analysis mechanism.

The above results indicate that the sensor with porous hydrogels effectively responds to pressure more than original hydrogels. Small pores (sub 10 μm level) are present in original and porous hydrogels. However, there is a visible difference between the two hydrogels at high pressure (Figure 4a,b). Original hydrogels with small pores are highly compact at low pressure, and consequently, the changes in conductive pathways are minimal under compression (Figure 4a). In contrast, porous hydrogels with both small and large pores (100–150 µm) allow more compaction and maintain high piezoresistivity due to forming more conductive pathways under increased pressure (Figure 4b). The structure of porous hydrogels with dense large-sized pores facilitates the closure of large pores at a high-pressure range, allowing the measurement of high pressures. 

The schematic pressure-sensing models of porous hydrogels sandwiched between two carbon cloth electrodes are illustrated in Figure 4c. The deformation of hydrogel B and the carbon cloth and the changing processes of the contact points under external force are also presented in Figure 4c. When low pressure (0.1–20 kPa) is applied to the sensor, the pores are squeezed to a high degree, forming many conductive paths in hydrogel B. Therefore, the output signal changes significantly, contributing to a high sensitivity of 12.7 kPa^−1^ in the low-pressure range. When pressure increases to a medium range, the deformation of the pores in hydrogels reaches saturation, while the contact area between the carbon cloth and hydrogels increases. At this stage, the decreasing rate in the total resistance of the sensor slows down, and the sensitivity decreases to 5.0 kPa^−1^ (20–50 kPa). A further increase in pressure compresses the carbon fiber in carbon cloth, thereby reducing the resistance. At this stage, the sensor only has a lower sensitivity of 3.2 kPa^−1^ in the pressure range of 50–100 kPa. 

### 3.4. Applications of the Sensor

**Figure 5:**
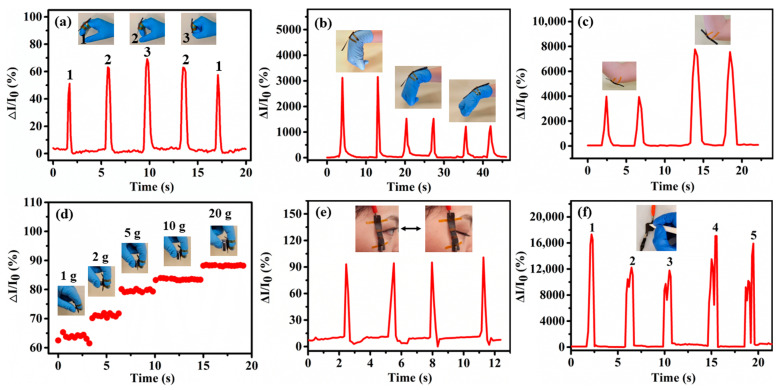
The signal responses arise from (**a**) finger bending, (**b**) wrist bending, and (**c**) elbow bending. (**d**) The signal responses of the sensors attached to the finger-holding standard weights of 1, 2, 5, 10, and 20 g. The signal responses from (**e**) eye blinking and (**f**) different handwriting.

The good performances of the proposed sensor have potential prospects in many application fields, including wearable electronic products and human activity monitoring systems. We further demonstrate that the sensor’s response based on hydrogel B is potentially useful in practical applications. The sensor is attached to each joint to monitor the finger (Figure 5a), wrist (Figure 5b), and elbow bending (Figure 5c). The signal waveform remains consistent under the same bending angle, while a significant change in current is observed with different joint-bending angles. The pressure produced by joint bending increases with the bending angle; therefore, when the bending angle increases, the sensor generates stronger signals, thus increasing the variation. The sensors are attached to the fingers to simulate touch sensation. Figure 5d shows that the sensor is attached to the index finger. The changes in pressure signals are observed when standard weights of 1, 2, 5, 10, and 20 g are held by hand in sequence. The sensor easily measures the force applied by the finger, suggesting that the sensor can play an important role in the tactile perception of the manipulator. Furthermore, the sensor can also monitor human microexpression attached near the eyes (Figure 5e), and the eye-opening/closing states are identified with good sensitivity. Interestingly, the sensors could distinguish different handwriting samples (Figure 5e). Due to the difference in writing power and direction, the handwriting samples of 1, 2, 3, 4, and 5 differ in number and peak shape, presenting an opportunity to realize handwriting anti-counterfeiting applications. Foot-stepping pressure is important information in biomechanics, healthcare, recovery, and diagnosis, especially for athletes who exercise extensively, teenagers in development, and patients suffering from Parkinson’s disease or diabetic foot ulcers [19]. Figure S4 (Supporting Information) shows the foot-stepping pressures detected by using the sensor. Because of the broad measuring range, the sensor can detect different stepping intensities, such as the weak step and the heavy step. The result demonstrates the sensor has potential applications in footwear electronics. 

**Figure 6:**
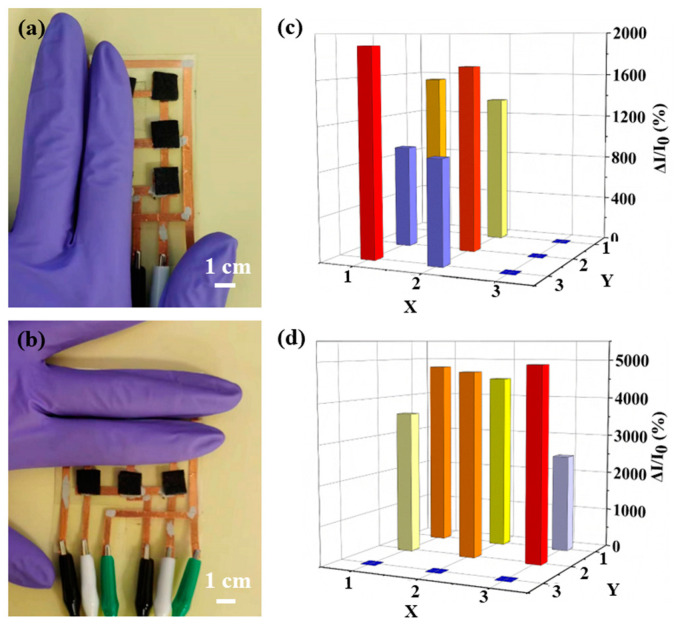
(**a**,**b**) Photograph of a sensor array (3 × 3 pixel) with different devices pressed by two fingers, and (**c**,**d**) and their corresponding signals.

An individual pressure sensor only provides limited information due to its low coverage area and integrated properties. Individual hydrogel B was integrated into a 3 × 3 pixel sensor array on a polyethylene terephthalate (PET) substrate to perceive the spatial distributions of pressure by employing silver glue to connect with the copper foil as the electrode (Figure 6). The 3 × 3 pixel sensor array was connected to a signal management circuit (Figure S5, Supporting Information), which included a micro-controller unit, an analog-to-digital converter (ADC), channel selection, and communication interface. Using the circuit diagram shown in Figure S5, the real-time signal can be transmitted to a computer via a communication interface. In this process, by applying pressure on the sensor array, the electrical resistance of the on-site sensor changes and the responsive current under applied pressure is recorded, generating the color contrast mapped with local pressure distribution. When two fingers are pressed on the sensor array (Figure 6a,b), the position and pressure of the finger are accurately determined through the responsive current changes (Figure 6c,d), consistent with the finger position. These results suggest the potential of the sensor arrays in E-skin devices for next-generation wearable electronics.

## 4. Conclusions

A simple, environment-friendly, low-cost, high-performance wearable piezoresistive pressure sensor has been developed using a highly porous LBG-based hydrogel. By tuning the porosity of porous LBG-based hydrogel (12.8%, 15.7%, 22.4%), the hydrogel with a porosity of 15.7% demonstrates excellent sensing performance. The porous LBG-based hydrogel offers a wide pressure detection range with high sensitivities (12.7, 5.0, and 3.2 kPa^−1^ under 0.1–20, 20–50, and 50–100 kPa), a fast response time (263 ms), and good durability over 500 loading/unloading cycles. These excellent properties are ascribed to the three-dimensional macroporous structure formed with dense large-sized pores (100–150 μm), facilitating the compression of large pores over a wide pressure range. Additionally, the sensor has promising practical applications in monitoring and identifying human activities. Their sensitivity and wide-range properties endow the porous LBG-based hydrogel with great potential for application in various wearable sensors. This work provides a good strategy for fabricating high-performance wearable pressure sensors in human motion monitoring with high sensitivity over a wide response range.

## Data Availability

The data are available upon reasonable request.

## Supplementary Materials

The following supporting information can be downloaded at: https://www.mdpi.com/article/10.3390/polym15122736/s1, Figure S1: SEM image of the commercial rosin particles; Figure S2: Cross-sectional SEM image of hydrogel C; Figure S3: Stability of the sensor with hydrogel B at 20 kPa for 500 cycles; Figure S4: Pressure signals when a foot steps on and steps off; Figure S5: A schematic diagram of a sensor array with a signal management circuit system; Table S1: Response time, detection limit, and durability of our sensor in comparison with other piezoresistive sensors based on hydrogels as reported in references.
