# Supplementary material for: Wearable Pressure Sensor Using Porous Natural Polymer Hydrogel Elastomers with High Sensitivity over a Wide Sensing Range
Source: Polymers (Basel). 2023 Jun 19;15(12):2736. doi: 10.3390/polym15122736 (PMC10301135; doi:10.3390/polym15122736)
Supplement: Supplementary file 1 [file polymers-15-02736-s001.zip › polymers-2428015-supplementary.pdf]

Supporting Information

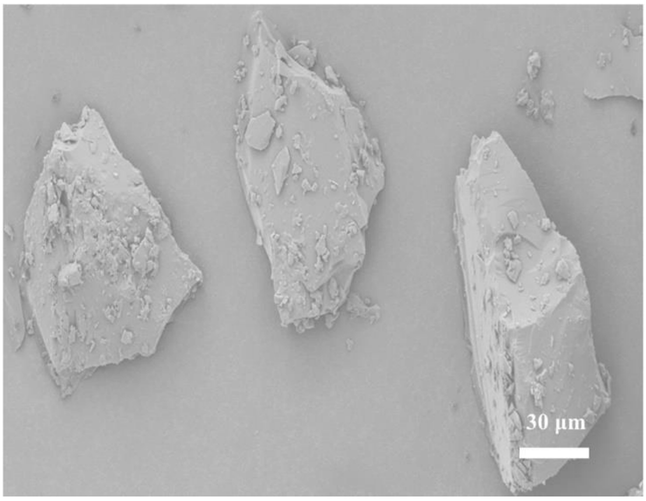

Figure S1 SEM image of the commercial rosin particles.

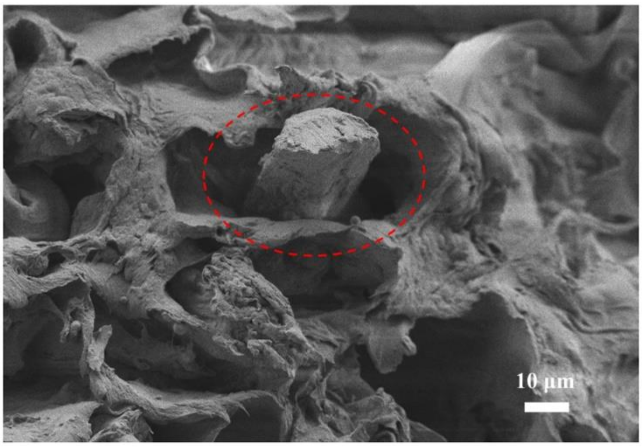

Figure S2 Cross-sectional SEM image of hydrogel C.

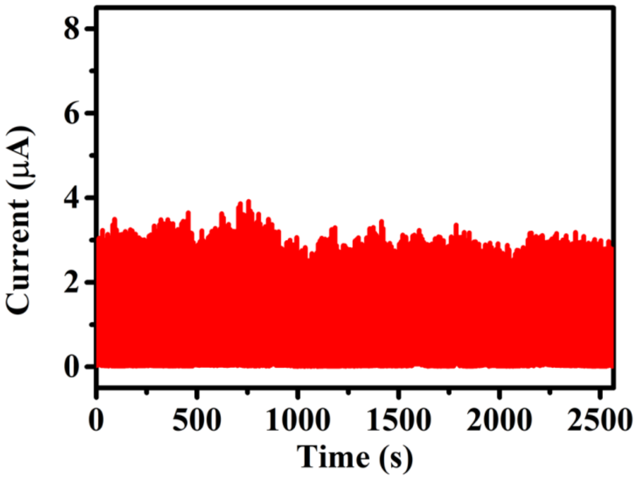

Figure S3 Stability of the sensor with hydrogel B at 20 kPa for 500 cycles.

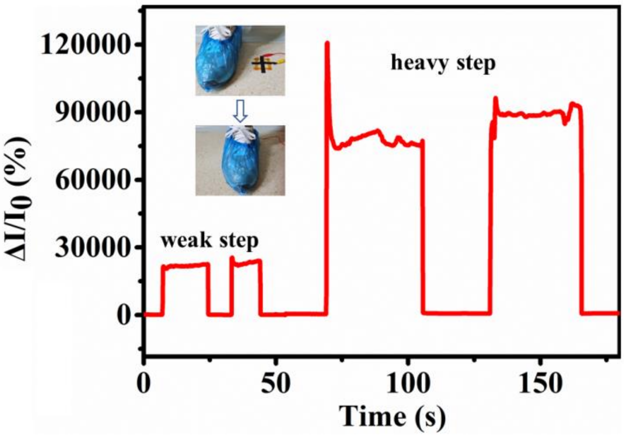

Figure S4. Pressure signals when a foot steps on and steps off.

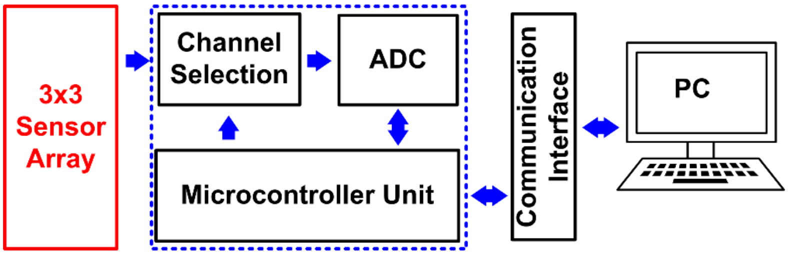

Figure S5. A schematic diagram of a sensor array with a signal management circuit system.

Table S1. Response time, detection limit, and durability of our sensor in comparison with other piezoresistive sensors based on hydrogels as reported in references.

	Response time	Detection limit	Durability
This work	263 ms	100 Pa	500 cylces
LBG-based hydrogels with double-rough surface[1]	356 ms		1000 cycles
MXene-composited PVA/polyvinyl pyrrolidone hydrogel [2]	33.5 ms	0.87 Pa	380 cycles
Gold-nanowire/polyacrylamide composite hydrogels[3]	0.5 s	0.2 Pa	10000 cycles
PVA-polyacrylamide hydrogel[4]	150 ms		500 cycles
CNTs/hydrophobically associated polyacrylamide hydrogel[5]	0.6 s		300 cycles

TEMPO-oxidized cellulose nanofibril/polypyrrole hydrogel[6]	< 10 ms	0.3 Pa	>9000 cycles

References

1. Huang, Y.; Liu, B.; Zhang, W.; Qu, G.; Jin, S.; Li, X.; Nie, Z.; Zhou, H. Highly Sensitive Active-powering Pressure Sensor Enabled by Integration of Double-rough Surface Hydrogel and Flexible Batteries. *npj Flexible Electronics* **2022**, *6*, 92.

2. Lu, Y.; Qu, X.; Zhao, W.; Ren, Y.; Si, W.; Wang, W.; Wang, Q.; Huang, W.; Dong, X. Highly Stretchable, Elastic, and Sensitive MXene-based Hydrogel for Flexible Strain and Pressure Sensors. *Research* **2020**, *1*, 2038560.

3. Yin, M.; Zhang, Y.; Yin, Z.; Zheng, Q.; Zhang, A. P. Micropatterned Elastic Gold-nanowire/polyacrylamide Composite Hydrogels for Wearable Pressure Sensors. *Adv. Mater. Technol.* **2018**, *3*, 1800051.

4. Ge, G.; Zhang, Y.; Shao, J.; Wang, W.; Si, W.; Huang, W.; Dong, X. Stretchable, Transparent, and Self-patterned Hydrogel-based Pressure Sensor for Human Motions Detection. *Adv. Funct. Mater.* **2018**, *28*, 1802576.

5. Qin, Z.; Sun, X.; Yu, Q.; Zhang, H.; Wu, X.; Yao, M.; Liu, W.; Yao, F.; Li, J. Carbon Nanotubes/Hydrophobically Associated Hydrogels as Ultrastretchable, Highly Sensitive, Stable Strain, and Pressure Sensors. *ACS Appl. Mater. Interfaces* **2020**, *12*, 4944-4953.

6. Gao, Y.; Liu, D.; Xie, Y.; Song, Y.; Zhu, E.; Shi, Z.; Yang, Q.; Xiong, C. Flexible and Sensitive Piezoresistive Electronic Skin Based on TOCN/PPy Hydrogel Films. *J Appl Polym Sci.* **2021**, *138*, 51367.
